# ﻿DNA Barcoding of Central European Gasteruptiidae and the rarely-collected families Evaniidae, Stephanidae, Trigonalidae, and Aulacidae (Hymenoptera, Apocrita)

**DOI:** 10.3897/zookeys.1189.114478

**Published:** 2024-01-17

**Authors:** Christian Schmid-Egger, Stefan Schmidt, Petr Bogusch

**Affiliations:** 1 Fischerstr. 1, 10317 Berlin, Germany Unaffiliated Berlin Germany; 2 SNSB-Zoologische Staatssammlung München, Munich, Germany SNSB-Zoologische Staatssammlung München Munich Germany; 3 University of Hradec Králové, Faculty of Science, Department of Biology, Hradec Králové, Czech Republic University of Hradec Králové Hradec Králové Czech Republic

**Keywords:** Central Europe, COI, DNA barcoding, insects, morphology, taxonomy

## Abstract

The study presents DNA barcoding results of five families of Hymenoptera in Germany. DNA barcodes are provided for 24 of the 25 species of *Gasteruption* occurring in Central Europe, including 18 of the 19 species recorded from Germany. The genetic diversity was higher than expected, with five species exhibiting two or more Barcode Index Number (BINs), whereas BIN sharing occurred in four species. *Gasteruptionfoveiceps* Semenov, 1892, **stat. nov.** is removed from synonymy with *G.nigrescens* Schletterer, 1885 and treated as a distinct species.

## ﻿Introduction

The present study provides the first attempt to compile a comprehensive DNA barcode library for Gasteruptiidae species recorded from Central Europe. We also included the barcodes of four species from the families Evaniidae, Stephanidae, Trigonalidae and Aulacidae. For practical reasons and because records from other countries of Central Europe are not available, we concentrate on the German species. The families Aulacidae, Evaniidae, Stephanidae and Trigonalidae are represented by a single or a few species in each family in Germany (Saure 2001).

The family Gasteruptiidae is represented in Europe by the single genus *Gasteruption*. The genus is represented in Central Europe with 25 species (Bogusch 2021). The majority of Central European species are predator-inquilines of various stem- and wood-nesting bee species (Apiformes), in particular of the genus *Hylaeus* (Colletidae). Several species attack nests of other bee species (families Megachilidae and Apidae) or, rarely, representatives of families Crabronidae and Vespidae (Wall 1994; Bogusch et al. 2018; Parslow et al. 2020; Bogusch 2021). Several species also parasitise soil-nesting bee species of the family Halictidae, such as *Gasteruptionhastator*, or nests of bees and wasps in vertical sand or loess walls (Parslow et al. 2020). The current taxonomy of this group was studied by van Achterberg and Talebi (2014) and Bogusch (2021), with the biology and host associations by Parslow et al. (2020).

The initial phase of the DNA barcoding projects focussed on species occurring in southern Germany, as part of the ‘Barcoding Fauna Bavarica’ project of the SNSB-Zoologische Staatssammlung München, Germany (ZSM). The project started in 2009 and aimed at assembling DNA barcodes for all Bavarian animal species (Hendrich et al. 2010; Hausmann et al. 2013). Since 2012, the ‘German Barcode of Life’ (GBOL) project added additional sequences. Previous barcode releases of Aculeata (Hymenoptera) dealt with the Anthophila or bees (Schmidt et al. 2015), the Spheciformes or digger wasps (Schmid‐Egger et al. 2019), the genus *Polistes* or paper wasps (Vespidae) (Schmid-Egger et al. 2017), and the remaining Vespoidea (Schmid-Egger and Schmidt 2021).

The present study focuses on Central European species of Gasteruptiidae, including 24 of the 25 species recorded from Central Europe, and three additional species from southern Europe. In addition, four species of the families Evaniidae, Stephanidae, Trigonalidae and Aulacidae are included. For detailed species numbers, see Table 1. Identification and taxonomy of species from Central Europe follow Bogusch (2021).

**Table 1.** *Gasteruption* species included in the present study showing their presence (+) in Central European countries (D = Germany, CZ = Czech Republic, SLO = Slovakia, A = Austria, H = Hungary, CH = Switzerland). All species known from Central Europe are considered in the table; some additional species from southern Europe are also analysed and mentioned here.

Species from Central Europe	D	CZ	SLO	A	H	CH	Notes
*Gasteruptionassectator* (Linnaeus, 1758)	+	+	+	+	+	+	See Fig. 1
*Gasteruptionboreale* (Thomson, 1883)	+	+	+	+	+		
*Gasteruptioncaucasicum* (Guérin-Méneville, 1844)	+	+	+	+	+	+	
*Gasteruptiondiversipes* (Abeille de Perrin, 1879)	+	+	+	+	+	+	
Gasteruption*dolichoderum* Schletterer, 1889							Not known from Central Europe
*Gasteruptionerythrostomum* (Dahlbom, 1831)	+	+	+	+	+	+	
*Gasteruptionforticorne* Semenov, 1892			+	+	+		
*Gasteruptionfoveiceps* Semenov, 1892							Not known from Central Europe
*Gasteruptionfreyi* (Tournier, 1877)	+	+	+	+	+	+	
*Gasteruptiongoberti* (Tournier, 1877)					+	+	
*Gasteruptionhastator* (Fabricius, 1804)	+	+	+	+	+	+	See Fig. 2
*Gasteruptionhungaricum* Szépligeti, 1895	+	+	+	+	+		
*Gasteruptioninsidiosum* Semenov, 1892			+		+		
*Gasteruptionjaculator* (Linnaeus, 1758)	+	+	+	+	+	+	
*Gasteruptionlaticeps* (Tournier, 1877)	+	+	+	+	+	+	
*Gasteruptionlugubre* Schletterer, 1889	+			+		+	Not available for study
*Gasteruptionmerceti* Kieffer, 1904	+	+	+	+	+	+	
*Gasteruptionminutum* (Tournier, 1877)	+	+	+	+		+	See Fig. 3
*Gasteruptionnigrescens* Schletterer, 1885	+	+	+	+	+	+	
*Gasteruptionnigritarse* (Thomson, 1883)	+	+	+	+	+		
*Gasteruptionopacum* (Tournier, 1877)	+	+	+	+	+	+	
*Gasteruptionpaternum* Schletterer, 1889		+	+	+	+	+	
*Gasteruptionphragmiticola* Saure, 2006	+	+	+		+		
*Gasteruptionschlettereri* Magretti, 1890							Not known from Central Europe
*Gasteruptionsubtile* Thomson, 1883	+	+	+	+	+	+	
*Gasteruptiontournieri* Schletterer, 1885	+	+	+	+	+	+	
*Gasteruptionundulatum* (Abeille de Perrin, 1879)	+	+	+	+	+	+	
*Gasteruptionvariolosum* (Abeille de Perrin, 1879)				+		+	
**Total**	**19**	**20**	**21**	**22**	**20**	**19**	

The barcoding projects were conducted in close cooperation with the Biodiversity Institute of Ontario, University of Guelph, within the framework of the International Barcode of Life initiative. All sequences and the associated project data are available through the Barcode of Life Data Systems (BOLD). The dataset includes mainly Central European specimens but covers additional specimens from the Mediterranean area.

## ﻿Materials and methods

### ﻿Sampling

The present study covers the family Gasteruptiidae from Germany and adjacent areas, with a single genus *Gasteruption*. The main source of material includes specimens from Central Europe deposited in the collections of the SNSB-Zoologische Staatssammlung München, Germany (**ZSM**), Biologiezentrum Linz, Austria (**OLL**) and the private collections of Christian Schmid-Egger (**CSE**) and Petr Bogusch (**PB**). Some specimens of Central European species were collected in countries other than Germany, mainly in northern Italy, because these species are rare or even close to extinction in Central Europe and, therefore, virtually impossible to obtain from this region. Specimens from the remaining families are deposited in the ZSM or the private collection of CSE.

Specimens were identified to species level using van Achterberg and Talebi (2014) and Bogusch (2021). A complete list of voucher specimens that were treated in the present study is given in Suppl. material 1.

### ﻿DNA sequencing

For DNA extraction, a single leg was removed from each specimen and sent to the Canadian Centre for DNA Barcoding (CCDB) in Guelph, Canada, for DNA extraction and barcode sequencing. DNA extraction, PCR amplification, and sequencing were conducted using standardised high-throughput protocols (Ivanova et al. 2006). The 658bp target region, starting from the 5’ end of the mitochondrial cytochrome *c* oxidase I (COI) gene, includes the DNA barcode region of the animal kingdom (Hebert et al. 2003). Specimens that were successfully sequenced are listed in Suppl. material 1, with sequence lengths and the number of unresolved bases. All specimen data are accessible in BOLD as a single citable dataset (dx.doi.org/10.5883/DS-GBGAST). The data include collecting locality, geographic coordinates, elevation, collector, one or more digital images, identifier, and voucher depository. Sequence data can be obtained through BOLD and include a detailed Laboratory Information Management System (LIMS) report, primer information, and access to trace files.

### ﻿Data analysis

We only analyse *Gasteruption* sequences here. Sequences of remaining Hymenoptera families are not shown here, but data are available in the BOLD system. Sequence divergence statistics were calculated using the Kimura two-parameter model of sequence evolution (Kimura 1980). Barcode Index Numbers (BINs) were assigned by the BOLD system, representing globally unique identifiers for clusters of sequences that correspond closely to biological species (Ratnasingham and Hebert 2013). For BIN assignment, a minimum sequence length of 500 bp is required, and sequences between 300 and 500 bp can join an existing BIN but will not create or split BINs. BINs provide an interim taxonomic system and a way to signify Molecular Taxonomic Units (MOTUs) prior to detailed taxonomic studies including morphology. Sequences were aligned using the BOLD Aligner (amino acid-based hidden Markov models). The analyses are based on sequences with a minimum length of 500 bp and <1% ambiguous bases. Genetic distances and summary statistics were calculated using analytical tools in BOLD and are given as mean and maximum pairwise distances for intraspecific variation and as minimum pairwise distances for interspecific variations.

### ﻿Species studied

All *Gasteruption* species known from Central Europe were studied (Table 1), based on information in Bogusch (2021), except *G.lugubre*, a very rare species known from the European Alps and some countries in southeast Europe to Turkey. Also, we added three species occurring in southern Europe (*G.dolichoderum*, *G.foveiceps* and *G.schlettereri*) for better comparison with similar species, and to assist in their identification. For other Hymenoptera families, see below.

## ﻿Results

For the present study, 152 sequences of 24 species of *Gasteruption* were analysed, with a length of at least 500 bp and less than 1% ambiguous bases. The dataset thus includes DNA barcodes of 24 of the 25 *Gasteruption* species known to occur in Central Europe (Table 1), including 18 of the 19 species recorded from Germany.

### ﻿Taxonomic treatment

DNA barcoding allows the identification of *Gasteruption* to the species level or, in a few cases, to the species group level because of BIN sharing. The species that exhibited BIN sharing or BIN divergence, or that are otherwise taxonomically challenging, are discussed below.

***Gasteruptionjaculator* (Linnaeus, 1758)**

*Gasteruptionjaculator* is widespread and common in Central Europe. The species exhibited BIN divergence, with a maximum intraspecific distance of 3.28% and separation into two different BINs. There are no morphological differences and no hint for species separation. The second BIN was also found in a single specimen from Slovakia, but generally, only a few specimens were examined. Further research is needed.

***Gasteruptionerythrostomum* (Dahlbom, 1831)**

*Gasteruptionerythrostomum* is widespread and common in Central Europe. The species exhibited BIN divergence, with a maximum intraspecific distance of 3.39% and separation into two different BINs. There are no morphological differences and no indication of the presence of separate species. One BIN was only found in two specimens from Bavaria (Germany), whereas the other BIN is widespread in the study area.

***Gasteruptionfoveiceps* Semenov, 1892, stat. nov.**

The present specimens of *Gasteruptionfoveiceps* originated from northern Italy (Aosta, Lombardy) and were formerly identified as *G.nigrescens* by CSE. However, a detailed examination by PB, considering the marked BIN difference in the species group, led to the discovery of different character states. Taking into account the morphological characters and synonyms of *G.nigriceps*, and the shape of the head and the shiny area between the antesternal and praepectal carinas (van Achterberg and Talebi 2014), the two specimens from Italy fit well with *G.foveiceps*. Thus, *G.foveiceps* is not a synonym of *G.nigrescens* but should be regarded as a valid species that occurs in southern Europe.

***Gasteruptionschlettereri* Magretti, 1890, *G.diversipes* (Abeille de Perrin, 1879) and *G.forticorne* Semenov, 1892**

Our original dataset includes altogether 14 specimens of this species complex, forming five clusters each with a BIN, but without a clear morphological distinction between *G.schletterei*, *G.forticorne* and *G.diversipes*. We cannot solve the taxonomic problems in this group with the few specimens at hand.

A single specimen of *G.diversipes* from Slovakia forms a cluster with specimens of *Gasteruptionschlettereri* from northern Italy and Croatia. They most probably belong to the same species, apart from the fact that the morphological characters of both taxa are different (identified by Cornelis van Achterberg and PB).

The other cluster comprises specimens from Hungary, Slovakia and Croatia, and agrees with *G.forticorne* by morphology. So, *G.forticorne* and “*G.diversipes*” can be identified in Central Europe with the key of Bogusch (2021) but the second taxon still has to be checked for the correct name. The situation in southern Europe is more complicated and needs final revision. The diagnostic characters for *G.forticorne*, like length of malar space, or colour of genitalia in *G.schlettereri* males, seem to be highly variable. Currently, European specimens identified as *G.schlettereri* probably belong to *G.diversipes* and *G.forticorne* and the real *G.schlettereri* occurs in the Middle East. Our present treatment is, therefore, provisional.

***Gasteruptionlaticeps* (Tournier, 1877)**

*Gasteruptionlaticeps* is widespread in Central Europe. The species exhibited BIN divergence, with a maximum intraspecific distance of 2.58% and separation into two different BINs. One BIN was recently found in eastern Central Europe and Greece (no records from Germany are available), and the other was found in southern France and the Aosta Valley in northern Italy. Probably it is a species separation in an eastern and southwestern distribution centre, as described in *Myrmosaatra* Panzer, 1801 or in the sibling species *Smicromyrmerufipes* (Fabricius, 1878) and *S.frankburgeri* Schmid-Egger, 2022 (Schmid-Egger and Schmidt 2021, 2022). The species group is in need of further investigation.

***Gasteruptionpaternum* Schletterer, 1889**

*Gasteruptionpaternum* is a rare species occurring in Central Europe, especially in the Pannonian lowlands, recorded from the following countries: Austria, Croatia, Czech Republic, France, mainland of Greece and Crete, Hungary, Italy, Slovakia and Switzerland. In all these countries, only a few specimens were recorded in the whole history of studies on this group (Bogusch 2021). The species exhibited BIN divergence, with a maximum intraspecific distance of 5.11% and separation into two different BINs. One BIN with one specimen originating from Tyrol in Austria, the other from the Italian Alps (Piedmont). The species complex needs further research.

***Gasteruptionassectator* (Linnaeus, 1758) aggregate**

Fig. 1

Johansson and van Achterberg (2016) revised the *Gasteruptionassectator* aggregate and resurrected two species, *G.boreale* and G. *nigritarse*, from synonymy. However, all three species remain difficult to identify by the morphology and the main morphological identification characters are continuous. Our results of the genetic barcoding showed no BIN divergence between the three taxa but some weak clustering into different clades of most (not all) examined species. This was also found in a study by Parslow et al. (2021), which also consistently recovered these species as one clade.

**Figure 1.**
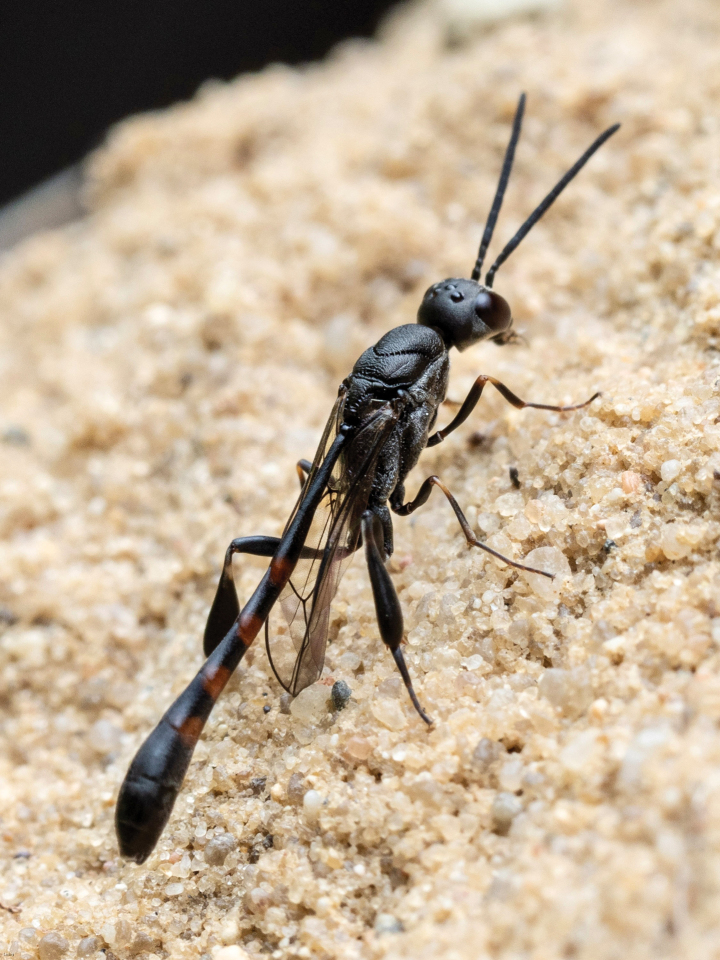
Male of *Gasteruptionassectator* from Saxony (Germany). (Photo W.H. Liebig).

Additionally, specimens from higher altitudes with differently sculptured mesonotum form a separate sister group to other barcoded specimens. This situation seems to indicate the presence of a new species, as discussed by van Achterberg and Talebi (2014). Further research is needed to assess if the *G.assectator* aggr. includes only one highly variable species or three or more valid and different species. The use of a nuclear gene may lead to more precise results, as shown by Praz et al. (2019) in bees of the *Andrenabicolor* species group.

***Gasteruptionhastator* (Fabricius, 1804)**

Fig. 2

*Gasteruptionhastator* is a widespread species of southern Central Europe and very common in southern Europe. The species exhibited BIN divergence, with a maximum intraspecific distance of 2.63% and separation into two different BINs. One BIN with one specimen origin from Aosta Valley in northwest Italy, the other from various locations. The species needs further research and may consist of a species complex, also seen under the impression of a very long list of synonyms (van Achterberg and Talebi 2014).

**Figure 2.**
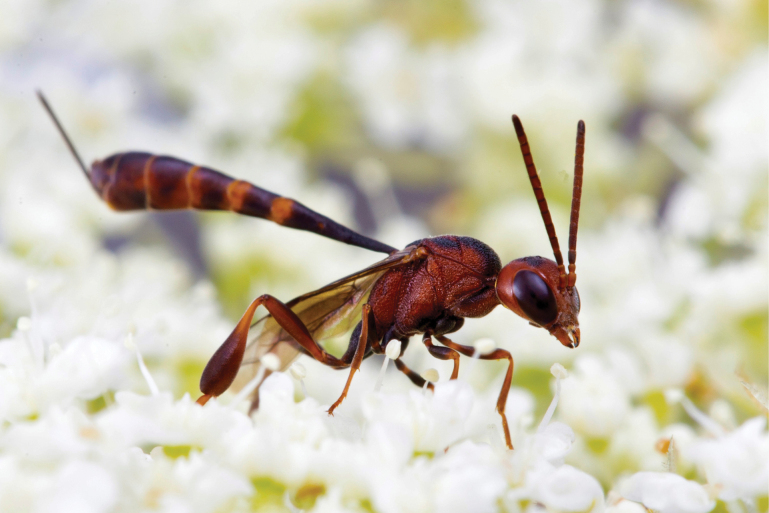
Female of *Gasteruptionhastator* from Saxony (Germany). It is the only largely red-coloured *Gasteruption* species in Central Europe. (Photo W.H. Liebig).

***Gasteruptioninsidiosum* Semenov, 1892**

*Gasteruptioninsidiosum* is a rare species of the Middle East, reaching East Europe with its north-western part of the distribution. The specimen from Turkey forms a sister group to the group of *G.erythrostomum*, *G.nigrescens*, *G.foveiceps* and *G.phragmiticola*, while the specimen from Slovakia is also part of this group, next to *G.erythrostomum*. Because the specimen from Slovakia differs in several characters from true *G.insidiosum* from Turkey, Greece and Bulgaria, it could be a separate species or belong to some of the synonyms of this species. However, the species descriptions are incomplete and short and some of the types are unavailable, so further research is needed to decide on the identification of the specimen from Slovakia.

**Figure 3.**
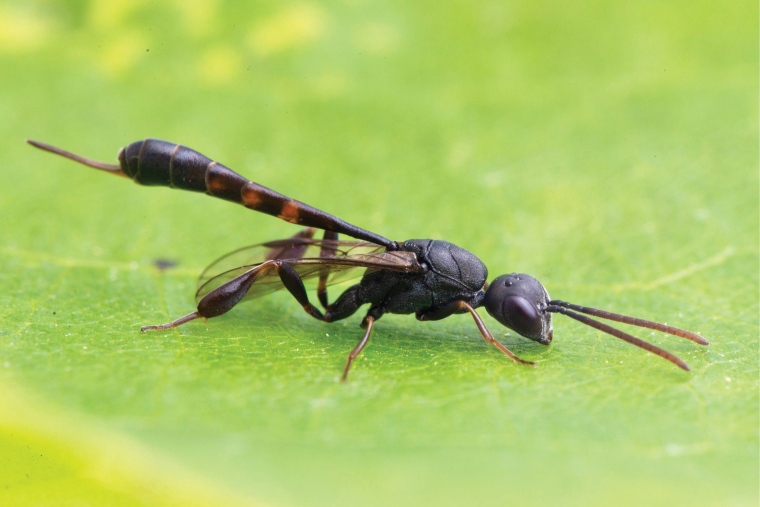
Female of *Gasteruptionminutum* from Saxony (Germany) (Photo W.H. Liebig).

## ﻿Discussion

### ﻿DNA barcoding of German species

For the present study, 18 of the 19 of the *Gasteruption* species that are known to occur in Germany were analysed by DNA barcoding. In two recent DNA barcoding studies dealing with German Apiformes (bees) (Schmidt et al. 2015) and German Spheciformes (Schmid-Egger et al. 2019, 88% of the German species were covered, although the number of German species is, with 584 species in bees and 273 species in Spheciformes, much larger compared to *Gasteruption*. For Europe, 25 of the 26 species recorded from Central Europe (Bogusch 2021) were available for study.

### ﻿BIN diversity

The most surprising result in this study is the unexpectedly high BIN diversity, suggesting a higher-than-expected species diversity. Five species exhibited two or more BINs. BIN sharing (i.e., two or more species that share the same BIN and are not separable by DNA barcoding) occurred in four species.

We refer to the discussion in Schmid-Egger and Schmidt (2021) on how to deal with and interpret the BIN diversity and problems with a lack of clear morphological characters. The present study confirms findings from previous studies that there are more genetically (BIN) based entities than morphological taxa in any examined Hymenoptera family. Although the BIN can change as more sequences are added, we suggest, whenever possible, to include the Barcode Index Number (BIN) in any further treatment of the species with BIN diversity, including a link to the BIN or the specimens in BOLD, in case the BINs represent distinct species.

### ﻿Other Hymenoptera families

The present study provided the opportunity to deal with some rare and poorly known Hymenoptera families with a reference to the German fauna. The families treated here include six species, five of which were DNA barcoded (Table 2). *Brachygasterminutus* (Evaniidae) is a parasitoid of cockroach ootheca (Blattoidea), and *Stephanusserrator* (Stephanidae) parasitises larva of longhorn beetles (Cerambycidae). *Pseudogonaloshahnii* (Trigonalidae) is a hyperparasitoid larva of Ichneumonoidea and can develop only when its egg is ingested by a parasitised caterpillar of an owlet moth (Erebidae and Noctuidae). Aulacidae are koinobiont endoparasitoids of wood-boring larvae of Xiphydriidae (Hymenoptera), Cerambycidae and Buprestidae (Coleoptera). The genus *Pristaulacus* was revised by Turrisi (2011). Distribution of the species is insufficiently known, but it can be assumed that most or all species are widespread. The species are rarely collected, with few specimens present in museum collections.

**Table 2.** Species of Hymenoptera families with a single, or very few, species from Germany that are included in the present study. For checklists of German species see Saure (2001); for *Pristaulacus*, also Turrisi (2011). All mentioned genera include only one species in Germany, apart from *Pristaulacus* with five German species (Turrisi 2011).

Family	Species
Evaniidae	*Brachygasterminutus* (Olivier, 1792)
Stephanidae	*Stephanusserrator* (Fabricius, 1798)
Trigonalidae	*Pseudogonaloshahnii* (Spinola, 1840)
Aulacidae	*Aulacusstriatus* Jurine, 1807
Aulacidae	*Pristaulacuscompressus* (Spinola, 1808)

## ﻿Additional information

### Author ORCIDs

Stefan Schmidt https://orcid.org/0000-0001-5751-8706

Petr Bogusch https://orcid.org/0000-0002-4554-6141

### Data availability

All of the data that support the findings of this study are available in the main text or Supplementary Information.

## Supplementary materials

Supplementary material 1 List of voucher specimens Data type pdf Explanation note List of voucher specimens with specimen ID, country of origin, collection date, specimen depository, Barcode Index Number (BIN) and sequencing success (COI-fragment length in bp, in square brackets number of unresolved bases). https://binary.pensoft.net/file/969033This dataset is made available under the Open Database License (http://opendatacommons.org/licenses/odbl/1.0/). The Open Database License (ODbL) is a license agreement intended to allow users to freely share, modify, and use this Dataset while maintaining this same freedom for others, provided that the original source and author(s) are credited.Christian Schmid-Egger, Stefan Schmidt, Petr Bogusch

Supplementary material 2 Barcoding statistics Data type pdf Explanation note Barcoding statistics with mean intraspecific distance, maximum intraspecific distance, nearest neighbour species, distance to nearest neighbour species, Barcode Index Number (BIN), country, and number of specimens. Asterisks indicate species with BIN sharing. https://binary.pensoft.net/file/969034This dataset is made available under the Open Database License (http://opendatacommons.org/licenses/odbl/1.0/). The Open Database License (ODbL) is a license agreement intended to allow users to freely share, modify, and use this Dataset while maintaining this same freedom for others, provided that the original source and author(s) are credited.Christian Schmid-Egger, Stefan Schmidt, Petr Bogusch
