# Supplementary material for: ﻿DNA Barcoding of Central European Gasteruptiidae and the rarely-collected families Evaniidae, Stephanidae, Trigonalidae, and Aulacidae (Hymenoptera, Apocrita)
Source: Zookeys. 2024 Jan 17;1189:275–86. doi: 10.3897/zookeys.1189.114478 (PMC10809266; doi:10.3897/zookeys.1189.114478)
Supplement: Supplementary material 1 — List of voucher specimens [file zookeys-1189-275_article-114478__-s001.pdf]

Supplement 1. List of voucher specimens with specimen ID, country of origin, collection date, specimen depository, Barcode Index Number (BIN) and sequencing success (COI-fragment length in bp, in square brackets number of unresolved bases).

Family	Species	Specimen ID	Country	Collection Date	Depository	BIN	COI-5P
Aulacidae	Aulacus striatus	BC ZSM HYM 10384	Netherlands	04-Jun-2010	SNSB, Zoologische Staatssammlung Muenchen	BOLD:ABU9057	658[0n]
Aulacidae	Aulacus striatus	BC ZSM HYM 10385	Netherlands	05-Jun-2010	SNSB, Zoologische Staatssammlung Muenchen	BOLD:ABU9057	658[0n]
Aulacidae	Aulacus striatus	BC ZSM HYM 10386	Netherlands	06-Jun-2010	SNSB, Zoologische Staatssammlung Muenchen	BOLD:ABU9057	658[0n]
Aulacidae	Aulacus striatus	BC ZSM HYM 10387	Netherlands	07-Jun-2010	SNSB, Zoologische Staatssammlung Muenchen	BOLD:ABU9057	658[0n]
Aulacidae	Pristaulacus compressus	GBOL12865	France	01-Jul-2013	SNSB, Zoologische Staatssammlung Muenchen	BOLD:ADA3999	658[0n]
Evaniidae	Brachygaster minutus	BC ZSM HYM 20591	Germany	09-Jun-2012	SNSB, Zoologische Staatssammlung Muenchen	BOLD:AAW7612	664[0n]
Evaniidae	Brachygaster minutus	BC ZSM HYM 20592	Germany	11-Jun-2011	SNSB, Zoologische Staatssammlung Muenchen	BOLD:AAW7612	664[0n]
Evaniidae	Brachygaster minutus	BC ZSM HYM 21375	Russia	24-Jul-2013	SNSB, Zoologische Staatssammlung Muenchen	BOLD:AAW7612	658[0n]
Gasteruptiidae	Gasteruption assectator	BC ZSM HYM 05081	Germany	23-Jun-2008	SNSB, Zoologische Staatssammlung Muenchen	BOLD:AAM3893	658[0n]
Gasteruptiidae	Gasteruption assectator	BC ZSM HYM 05082	Germany	17-Jun-2002	SNSB, Zoologische Staatssammlung Muenchen	BOLD:AAM3893	658[0n]
Gasteruptiidae	Gasteruption assectator	BC ZSM HYM 05083	Germany	08-Jun-2003	SNSB, Zoologische Staatssammlung Muenchen	BOLD:AAM3893	658[0n]
Gasteruptiidae	Gasteruption assectator	BC ZSM HYM 05084	Germany	28-Jul-2005	SNSB, Zoologische Staatssammlung Muenchen	BOLD:AAM3893	658[0n]
Gasteruptiidae	Gasteruption assectator	BC ZSM HYM 13915	Germany	28-Jul-2005	SNSB, Zoologische Staatssammlung Muenchen	BOLD:AAM3893	658[0n]
Gasteruptiidae	Gasteruption assectator	BC ZSM HYM 13916	Germany	08-Jun-2003	SNSB, Zoologische Staatssammlung Muenchen	BOLD:AAM3893	658[0n]
Gasteruptiidae	Gasteruption assectator	BC ZSM HYM 13917	Germany	15-Jul-2008	SNSB, Zoologische Staatssammlung Muenchen	BOLD:AAM3893	658[0n]
Gasteruptiidae	Gasteruption assectator	BC ZSM HYM 13918	Germany	27-Jul-2006	SNSB, Zoologische Staatssammlung Muenchen	BOLD:AAM3893	658[0n]
Gasteruptiidae	Gasteruption assectator	BC ZSM HYM 13919	Germany	27-Jul-2006	SNSB, Zoologische Staatssammlung Muenchen	BOLD:AAM3893	658[0n]
Gasteruptiidae	Gasteruption assectator	BC ZSM HYM 13920	Germany	17-Jun-2002	SNSB, Zoologische Staatssammlung Muenchen	BOLD:AAM3893	658[0n]
Gasteruptiidae	Gasteruption assectator	BC ZSM HYM 13921	Germany	17-Jun-2002	SNSB, Zoologische Staatssammlung Muenchen	BOLD:AAM3893	658[0n]
Gasteruptiidae	Gasteruption assectator	BC ZSM HYM 18390	France	10-Jul-2010	SNSB, Zoologische Staatssammlung Muenchen	BOLD:AAM3893	658[0n]
Gasteruptiidae	Gasteruption assectator	BC ZSM HYM 18391	France	12-Jul-2009	SNSB, Zoologische Staatssammlung Muenchen	BOLD:AAM3893	658[0n]
Gasteruptiidae	Gasteruption assectator	BC ZSM HYM 18393	Germany	22-Jul-2009	SNSB, Zoologische Staatssammlung Muenchen	BOLD:AAM3893	658[0n]
Gasteruptiidae	Gasteruption assectator	BC ZSM HYM 18394	Germany	22-Jul-2009	SNSB, Zoologische Staatssammlung Muenchen	BOLD:AAM3893	658[0n]
Gasteruptiidae	Gasteruption assectator	BC ZSM HYM 20588	Germany	01-Jul-2012	SNSB, Zoologische Staatssammlung Muenchen	BOLD:AAM3893	664[0n]
Gasteruptiidae	Gasteruption assectator	ZSM-HYM-34400-A01	Hungary	15-Jul-2015	Private Collection of Petr Bogusch	BOLD:AAM3893	505[0n]
Gasteruptiidae	Gasteruption assectator	ZSM-HYM-34400-A03	Slovakia	28-Jun-2005	Private Collection of Petr Bogusch	BOLD:AAM3893	565[0n]
Gasteruptiidae	Gasteruption assectator	ZSM-HYM-34400-H09	Slovakia	07-Jun-2018	Private Collection of Petr Bogusch	BOLD:AAM3893	620[0n]
Gasteruptiidae	Gasteruption boreale	ZSM-HYM-34400-B02	Czech Republic	14-Aug-2018	Private Collection of Petr Bogusch	BOLD:AAM3893	567[0n]
Gasteruptiidae	Gasteruption boreale	ZSM-HYM-34400-B03	Czech Republic	14-Jun-2019	Private Collection of Petr Bogusch	BOLD:AAM3893	629[0n]
Gasteruptiidae	Gasteruption boreale	ZSM-HYM-34400-B04	Slovakia	22-Jul-2017	Private Collection of Petr Bogusch	BOLD:AAM3893	534[0n]
Gasteruptiidae	Gasteruption boreale	ZSM-HYM-34400-B05	Czech Republic	26-Jun-2019	Private Collection of Petr Bogusch	BOLD:AAM3893	625[0n]
Gasteruptiidae	Gasteruption boreale	ZSM-HYM-34400-H10	Czech Republic	15-Jun-2019	Private Collection of Petr Bogusch	BOLD:AAM3893	623[0n]
Gasteruptiidae	Gasteruption caucasicum	BC ZSM HYM 05097	Germany	22-Jun-2008	SNSB, Zoologische Staatssammlung Muenchen	BOLD:AAM4594	658[0n]
Gasteruptiidae	Gasteruption caucasicum	BC ZSM HYM 05098	Germany	16-Jul-2009	SNSB, Zoologische Staatssammlung Muenchen	BOLD:AAM4594	658[0n]
Gasteruptiidae	Gasteruption caucasicum	BC ZSM HYM 05099	Germany	10-Jun-2008	SNSB, Zoologische Staatssammlung Muenchen	BOLD:AAM4594	658[0n]
Gasteruptiidae	Gasteruption caucasicum	BC ZSM HYM 18410	Italy	11-Jul-2009	SNSB, Zoologische Staatssammlung Muenchen	BOLD:AAM4594	658[0n]
Gasteruptiidae	Gasteruption caucasicum	BC ZSM HYM 18411	Italy	27-Jul-2007	SNSB, Zoologische Staatssammlung Muenchen	BOLD:AAM4594	658[0n]
Gasteruptiidae	Gasteruption caucasicum	ZSM-HYM-34400-E08	Slovakia	17-Jun-2016	Private Collection of Petr Bogusch	BOLD:AAM4594	633[0n]
Gasteruptiidae	Gasteruption caucasicum	ZSM-HYM-34400-E09	Hungary	23-Jul-2017	Private Collection of Petr Bogusch	BOLD:AAM4594	628[0n]

Family	Species	Specimen ID	Country	Collection Date	Depository	BIN	COI-5P
Gasteruptiidae	Gasteruption caucasicum	ZSM-HYM-34400-E10	Croatia	08-Jul-2017	Private Collection of Petr Bogusch	BOLD:AAM4594	625[0n]
Gasteruptiidae	Gasteruption caucasicum	ZSM-HYM-34400-E11	Czech Republic	25-Jun-2019	Private Collection of Petr Bogusch	BOLD:AAM4594	636[0n]
Gasteruptiidae	Gasteruption diversipes	ZSM-HYM-34400-F04	Slovakia	02-Jul-2017	Private Collection of Petr Bogusch	BOLD:AEO5656	421[0n]
Gasteruptiidae	Gasteruption dolichoderum	BC ZSM HYM 16202	Turkey	20-Jul-1998	SNSB, Zoologische Staatssammlung Muenchen	BOLD:ACE0224	658[1n]
Gasteruptiidae	Gasteruption dolichoderum	ZSM-HYM-34400-C02	Cyprus	12-Aug-2019	Private Collection of Petr Bogusch	BOLD:ACE0224	634[0n]
Gasteruptiidae	Gasteruption dolichoderum	ZSM-HYM-34400-D12	Cyprus	09-Aug-2019	Private Collection of Petr Bogusch	BOLD:ACE0224	623[0n]
Gasteruptiidae	Gasteruption erythrostomum	BC ZSM HYM 05090	Germany	23-Jun-2008	SNSB, Zoologische Staatssammlung Muenchen	BOLD:AAN0587	600[0n]
Gasteruptiidae	Gasteruption erythrostomum	BC ZSM HYM 13209	Germany	05-Jul-2009	SNSB, Zoologische Staatssammlung Muenchen	BOLD:AEO6078	601[0n]
Gasteruptiidae	Gasteruption erythrostomum	BC ZSM HYM 13210	Germany	22-Jul-2010	SNSB, Zoologische Staatssammlung Muenchen	BOLD:AEO6078	638[0n]
Gasteruptiidae	Gasteruption erythrostomum	BC ZSM HYM 13211	Germany	19-Jun-2007	SNSB, Zoologische Staatssammlung Muenchen	BOLD:AEO6078	658[0n]
Gasteruptiidae	Gasteruption erythrostomum	BC ZSM HYM 16213	Germany	01-Jul-2008	SNSB, Zoologische Staatssammlung Muenchen	BOLD:AAN0587	610[3n]
Gasteruptiidae	Gasteruption erythrostomum	BC ZSM HYM 16214	Austria	02-Jul-2006	SNSB, Zoologische Staatssammlung Muenchen	BOLD:AEO6078	582[2n]
Gasteruptiidae	Gasteruption erythrostomum	ZSM-HYM-34400-C05	Germany	31-May-2011	Private Collection of Petr Bogusch	BOLD:AEO6078	421[0n]
Gasteruptiidae	Gasteruption erythrostomum	ZSM-HYM-34400-C06	Czech Republic	05-Jul-2018	Private Collection of Petr Bogusch	BOLD:AEO6078	470[0n]
Gasteruptiidae	Gasteruption erythrostomum	ZSM-HYM-34400-C07	Czech Republic	12-Jul-2020	Private Collection of Petr Bogusch	BOLD:AEO6078	513[0n]
Gasteruptiidae	Gasteruption forticorne	ZSM-HYM-34400-F01	Slovakia	16-Jun-2007	Private Collection of Petr Bogusch	BOLD:AED0665	384[0n]
Gasteruptiidae	Gasteruption forticorne	ZSM-HYM-34400-F02	Hungary	26-Jul-2017	Private Collection of Petr Bogusch	BOLD:AED0665	633[0n]
Gasteruptiidae	Gasteruption forticorne	ZSM-HYM-34400-G12	Croatia	08-Sep-2008	Oberoesterreichisches Landesmuseum, Biologiezentrum, Linz	BOLD:AED0665	634[0n]
Gasteruptiidae	Gasteruption foveiceps	BC ZSM HYM 05086	Italy	07-Jul-1995	SNSB, Zoologische Staatssammlung Muenchen	BOLD:AAM4590	534[0n]
Gasteruptiidae	Gasteruption foveiceps	BC ZSM HYM 16211	Italy	09-Jul-2006	SNSB, Zoologische Staatssammlung Muenchen	BOLD:AAM4590	658[0n]
Gasteruptiidae	Gasteruption freyi	ZSM-HYM-34400-B07	Czech Republic	15-Jun-2019	Private Collection of Petr Bogusch	BOLD:AEN9285	628[0n]
Gasteruptiidae	Gasteruption freyi	ZSM-HYM-34400-C04	Austria	17-Jul-2015	Private Collection of Petr Bogusch	BOLD:AEN9285	566[0n]
Gasteruptiidae	Gasteruption goberti	ZSM-HYM-34400-F07	Greece	03-Jul-2019	Private Collection of Petr Bogusch	BOLD:AEO5240	636[0n]
Gasteruptiidae	Gasteruption goberti	ZSM-HYM-34400-H04	Turkey	06-Jul-2006	Oberoesterreichisches Landesmuseum, Biologiezentrum, Linz	BOLD:AEO5240	633[0n]
Gasteruptiidae	Gasteruption hastator	BC ZSM HYM 16218	Italy	27-Jun-1999	SNSB, Zoologische Staatssammlung Muenchen	BOLD:AEZ6954	658[0n]
Gasteruptiidae	Gasteruption hastator	BC ZSM HYM 16220	France	23-May-1998	SNSB, Zoologische Staatssammlung Muenchen	BOLD:AAV6833	623[0n]
Gasteruptiidae	Gasteruption hastator	ZSM-HYM-34400-B10	Hungary	30-Jul-2018	Private Collection of Petr Bogusch	BOLD:AAV6833	629[0n]
Gasteruptiidae	Gasteruption hastator	ZSM-HYM-34400-B11	Slovakia	22-Jul-2017	Private Collection of Petr Bogusch	BOLD:AAV6833	634[1n]
Gasteruptiidae	Gasteruption hastator	ZSM-HYM-34400-B12	Hungary	23-Jul-2017	Private Collection of Petr Bogusch	BOLD:AAV6833	628[0n]
Gasteruptiidae	Gasteruption hastator	ZSM-HYM-34400-C01	Czech Republic	26-Jun-2019	Private Collection of Petr Bogusch	BOLD:AAV6833	629[1n]
Gasteruptiidae	Gasteruption hungaricum	ZSM-HYM-34400-F08	Hungary	26-Jun-2017	Private Collection of Petr Bogusch	BOLD:AEO6155	634[1n]
Gasteruptiidae	Gasteruption hungaricum	ZSM-HYM-34400-F10	Slovakia	14-Jun-2016	Private Collection of Petr Bogusch	BOLD:AEO6155	622[1n]
Gasteruptiidae	Gasteruption insidiosum	ZSM-HYM-34400-C10	Slovakia	21-Jul-2021	Private Collection of Petr Bogusch	BOLD:AEO1543	621[0n]
Gasteruptiidae	Gasteruption insidiosum	ZSM-HYM-34400-H05	Turkey	07-Jul-2006	Oberoesterreichisches Landesmuseum, Biologiezentrum, Linz	BOLD:AEO1542	635[0n]
Gasteruptiidae	Gasteruption jaculator	BC ZSM HYM 05096	Germany	10-Jun-2008	SNSB, Zoologische Staatssammlung Muenchen	BOLD:AAN0586	658[0n]
Gasteruptiidae	Gasteruption jaculator	BC ZSM HYM 13212	Germany	26-Jul-2004	SNSB, Zoologische Staatssammlung Muenchen	BOLD:AAN0586	658[0n]
Gasteruptiidae	Gasteruption jaculator	BC ZSM HYM 13213	Germany	15-Jul-2008	SNSB, Zoologische Staatssammlung Muenchen	BOLD:AAN0586	658[0n]
Gasteruptiidae	Gasteruption jaculator	BC ZSM HYM 13214	Germany	22-Jun-2002	SNSB, Zoologische Staatssammlung Muenchen	BOLD:AAN0586	658[0n]
Gasteruptiidae	Gasteruption jaculator	ZSM-HYM-34400-D06	Czech Republic	12-Aug-2018	Private Collection of Petr Bogusch		132[0n]
Gasteruptiidae	Gasteruption jaculator	ZSM-HYM-34400-D07	Slovakia	07-Jun-2018	Private Collection of Petr Bogusch		290[0n]

Family	Species	Specimen ID	Country	Collection Date	Depository	BIN	COI-5P
Gasteruptiidae	Gasteruption laticeps	BC ZSM HYM 18407	France	10-Jul-2010	SNSB, Zoologische Staatssammlung Muenchen	BOLD:ACH3937	658[0n]
Gasteruptiidae	Gasteruption laticeps	BC ZSM HYM 18408	France	12-Jul-2009	SNSB, Zoologische Staatssammlung Muenchen	BOLD:ACH3937	658[0n]
Gasteruptiidae	Gasteruption laticeps	BC ZSM HYM 18409	Italy	25-Jul-1999	SNSB, Zoologische Staatssammlung Muenchen	BOLD:ACH3937	606[0n]
Gasteruptiidae	Gasteruption laticeps	ZSM-HYM-34400-E12	Greece	03-Jul-2019	Private Collection of Petr Bogusch	BOLD:AAU2086	636[0n]
Gasteruptiidae	Gasteruption laticeps	ZSM-HYM-34400-F11	Slovakia	07-Jun-2018	Private Collection of Petr Bogusch	BOLD:AAU2086	621[0n]
Gasteruptiidae	Gasteruption laticeps	ZSM-HYM-34400-F12	Hungary	06-Jan-2018	Private Collection of Petr Bogusch	BOLD:AAU2086	636[0n]
Gasteruptiidae	Gasteruption laticeps	ZSM-HYM-34400-G01	Czech Republic	27-Jan-2018	Private Collection of Petr Bogusch	BOLD:AAU2086	635[0n]
Gasteruptiidae	Gasteruption laticeps	ZSM-HYM-34400-G02	Czech Republic	15-Jul-2018	Private Collection of Petr Bogusch	BOLD:AAU2086	635[0n]
Gasteruptiidae	Gasteruption laticeps	ZSM-HYM-34400-G03	Czech Republic	01-Aug-2019	Private Collection of Petr Bogusch	BOLD:AAU2086	602[0n]
Gasteruptiidae	Gasteruption laticeps	ZSM-HYM-34400-H07	Czech Republic	25-Jun-2019	Private Collection of Petr Bogusch	BOLD:AAU2086	629[0n]
Gasteruptiidae	Gasteruption merceti	BC ZSM HYM 16216	Italy	11-Jul-2009	SNSB, Zoologische Staatssammlung Muenchen	BOLD:ACE0348	658[0n]
Gasteruptiidae	Gasteruption merceti	BC ZSM HYM 16217	Italy	25-Jul-1999	SNSB, Zoologische Staatssammlung Muenchen	BOLD:ACE0348	658[0n]
Gasteruptiidae	Gasteruption merceti	BC ZSM HYM 18417	Italy	13-May-2006	SNSB, Zoologische Staatssammlung Muenchen	BOLD:ACE0348	658[0n]
Gasteruptiidae	Gasteruption merceti	ZSM-HYM-34400-D08	Czech Republic	15-Jul-2018	Private Collection of Petr Bogusch	BOLD:ACE0348	637[0n]
Gasteruptiidae	Gasteruption merceti	ZSM-HYM-34400-D09	Slovakia	19-Jun-2016	Private Collection of Petr Bogusch	BOLD:ACE0348	636[0n]
Gasteruptiidae	Gasteruption merceti	ZSM-HYM-34400-D10	Italy	28-Jun-2017	Private Collection of Petr Bogusch	BOLD:ACE0348	637[0n]
Gasteruptiidae	Gasteruption merceti	ZSM-HYM-34400-D11	Serbia	05-May-2018	Private Collection of Petr Bogusch	BOLD:ACE0348	624[0n]
Gasteruptiidae	Gasteruption minutum	BC ZSM HYM 16221	France	09-Jul-2010	SNSB, Zoologische Staatssammlung Muenchen	BOLD:ACE0418	658[0n]
Gasteruptiidae	Gasteruption minutum	BC ZSM HYM 16222	Italy	13-Aug-2008	SNSB, Zoologische Staatssammlung Muenchen	BOLD:ACE0418	658[0n]
Gasteruptiidae	Gasteruption minutum	BC ZSM HYM 16223	Italy	09-Jul-2006	SNSB, Zoologische Staatssammlung Muenchen	BOLD:ACE0418	595[1n]
Gasteruptiidae	Gasteruption minutum	ZSM-HYM-34400-B06	Slovakia	02-Jun-2017	Private Collection of Petr Bogusch	BOLD:ACE0418	609[0n]
Gasteruptiidae	Gasteruption minutum	ZSM-HYM-34400-B08	Czech Republic	09-Jun-2019	Private Collection of Petr Bogusch	BOLD:ACE0418	589[0n]
Gasteruptiidae	Gasteruption minutum	ZSM-HYM-34400-B09	Czech Republic	12-Jul-2020	Private Collection of Petr Bogusch	BOLD:ACE0418	628[0n]
Gasteruptiidae	Gasteruption nigrescens	BC ZSM HYM 05085	Germany	21-May-2007	SNSB, Zoologische Staatssammlung Muenchen	BOLD:AAM4589	658[0n]
Gasteruptiidae	Gasteruption nigrescens	BC ZSM HYM 05087	Germany	22-Jul-2004	SNSB, Zoologische Staatssammlung Muenchen	BOLD:AAM4589	658[0n]
Gasteruptiidae	Gasteruption nigrescens	ZSM-HYM-34400-C03	Slovakia	07-Jun-2018	Private Collection of Petr Bogusch	BOLD:AAM4589	469[0n]
Gasteruptiidae	Gasteruption nigrescens	ZSM-HYM-34400-D03	Czech Republic	14-Jun-2019	Private Collection of Petr Bogusch	BOLD:AAM4589	620[0n]
Gasteruptiidae	Gasteruption nigrescens	ZSM-HYM-34400-D04	Slovakia	15-Jul-2016	Private Collection of Petr Bogusch	BOLD:AAM4589	585[0n]
Gasteruptiidae	Gasteruption nigrescens	ZSM-HYM-34400-D05	Slovakia	27-Jun-2010	Private Collection of Petr Bogusch		145[0n]
Gasteruptiidae	Gasteruption nigritarse	ZSM-HYM-34400-A04	Czech Republic	03-Jun-2019	Private Collection of Petr Bogusch	BOLD:AAM3893	629[0n]
Gasteruptiidae	Gasteruption nigritarse	ZSM-HYM-34400-A05	Czech Republic	16-Jul-2017	Private Collection of Petr Bogusch	BOLD:AAM3893	626[0n]
Gasteruptiidae	Gasteruption nigritarse	ZSM-HYM-34400-A06	Slovakia	22-Jul-2017	Private Collection of Petr Bogusch	BOLD:AAM3893	608[0n]
Gasteruptiidae	Gasteruption nigritarse	ZSM-HYM-34400-A07	Czech Republic	14-Aug-2017	Private Collection of Petr Bogusch	BOLD:AAM3893	626[0n]
Gasteruptiidae	Gasteruption nigritarse	ZSM-HYM-34400-A08	Czech Republic	15-Jul-2016	Private Collection of Petr Bogusch	BOLD:AAM3893	627[0n]
Gasteruptiidae	Gasteruption nigritarse	ZSM-HYM-34400-A09	Hungary	24-Jul-2017	Private Collection of Petr Bogusch	BOLD:AAM3893	627[0n]
Gasteruptiidae	Gasteruption nigritarse	ZSM-HYM-34400-A10	Czech Republic	25-Jun-2019	Private Collection of Petr Bogusch	BOLD:AAM3893	531[0n]
Gasteruptiidae	Gasteruption opacum	BC ZSM HYM 18404	Italy	17-Jun-2009	SNSB, Zoologische Staatssammlung Muenchen	BOLD:ACH4090	658[0n]
Gasteruptiidae	Gasteruption opacum	BC ZSM HYM 18405	Italy	09-Jul-2006	SNSB, Zoologische Staatssammlung Muenchen	BOLD:ACH4090	658[0n]
Gasteruptiidae	Gasteruption opacum	ZSM-HYM-34400-E01	Italy	16-Jun-2017	Private Collection of Petr Bogusch	BOLD:ACH4090	636[0n]
Gasteruptiidae	Gasteruption opacum	ZSM-HYM-34400-E02	Hungary	24-Jul-2017	Private Collection of Petr Bogusch	BOLD:ACH4090	635[0n]
Gasteruptiidae	Gasteruption opacum	ZSM-HYM-34400-E03	Croatia	08-Jul-2017	Private Collection of Petr Bogusch	BOLD:ACH4090	635[0n]
Gasteruptiidae	Gasteruption opacum	ZSM-HYM-34400-E04	Slovakia	08-Jun-2017	Private Collection of Petr Bogusch	BOLD:ACH4090	596[1n]

Family	Species	Specimen ID	Country	Collection Date	Depository	BIN	COI-5P
Gasteruptiidae	Gasteruption opacum	ZSM-HYM-34400-F06	Croatia	08-Jul-2017	Private Collection of Petr Bogusch	BOLD:ACH4090	635[0n]
Gasteruptiidae	Gasteruption paternum	BC ZSM HYM 16205	Austria	02-Jul-2006	SNSB, Zoologische Staatssammlung Muenchen	BOLD:ACE0475	658[0n]
Gasteruptiidae	Gasteruption paternum	BC ZSM HYM 16206	Italy	11-Jul-2009	SNSB, Zoologische Staatssammlung Muenchen	BOLD:ACE0342	658[0n]
Gasteruptiidae	Gasteruption paternum	BC ZSM HYM 16207	Italy	11-Jul-2009	SNSB, Zoologische Staatssammlung Muenchen	BOLD:ACE0342	658[0n]
Gasteruptiidae	Gasteruption phragmiticola	BC ZSM HYM 05088	Germany	22-Jul-2004	SNSB, Zoologische Staatssammlung Muenchen	BOLD:AAM5488	658[0n]
Gasteruptiidae	Gasteruption phragmiticola	BC ZSM HYM 05089	Germany	22-Jul-2004	SNSB, Zoologische Staatssammlung Muenchen	BOLD:AAM5488	658[0n]
Gasteruptiidae	Gasteruption phragmiticola	ZSM-HYM-34400-C11	Czech Republic	20-Feb-2018	Private Collection of Petr Bogusch	BOLD:AAM5488	624[0n]
Gasteruptiidae	Gasteruption phragmiticola	ZSM-HYM-34400-C12	Hungary	11-Jan-2017	Private Collection of Petr Bogusch	BOLD:AAM5488	624[0n]
Gasteruptiidae	Gasteruption phragmiticola	ZSM-HYM-34400-D01	Hungary	15-Jul-2016	Private Collection of Petr Bogusch	BOLD:AAM5488	628[0n]
Gasteruptiidae	Gasteruption phragmiticola	ZSM-HYM-34400-D02	Czech Republic	06-Mar-2017	Private Collection of Petr Bogusch	BOLD:AAM5488	625[0n]
Gasteruptiidae	Gasteruption phragmiticola	ZSM-HYM-34400-H11	Hungary	30-Jul-2018	Private Collection of Petr Bogusch	BOLD:AAM5488	632[0n]
Gasteruptiidae	Gasteruption schlettereri	ZSM-HYM-34400-G06	Italy	13-Jun-2017	Private Collection of Petr Bogusch	BOLD:AEO5656	572[1n]
Gasteruptiidae	Gasteruption schlettereri	ZSM-HYM-34400-G08	Croatia	08-Sep-2008	Private Collection of Petr Bogusch	BOLD:AEO5656	421[0n]
Gasteruptiidae	Gasteruption schlettereri	ZSM-HYM-34400-H06	Italy	09-Aug-2014	Private Collection of Jan Ruzicka	BOLD:AEO5656	632[0n]
Gasteruptiidae	Gasteruption subtile	ZSM-HYM-34400-G04	Slovakia	04-Aug-2008	Private Collection of Petr Bogusch	BOLD:ADD2731	338[1n]
Gasteruptiidae	Gasteruption subtile	ZSM-HYM-34400-G05	Slovakia	20-Jun-2008	Private Collection of Petr Bogusch	BOLD:ADD2731	371[0n]
Gasteruptiidae	Gasteruption tournieri	BC ZSM HYM 05106	Germany	28-Jul-2005	SNSB, Zoologische Staatssammlung Muenchen	BOLD:AAM3895	658[0n]
Gasteruptiidae	Gasteruption tournieri	BC ZSM HYM 05107	Germany	22-Jul-2004	SNSB, Zoologische Staatssammlung Muenchen	BOLD:AAM3895	658[0n]
Gasteruptiidae	Gasteruption tournieri	BC ZSM HYM 05108	Germany	24-Jul-2008	SNSB, Zoologische Staatssammlung Muenchen	BOLD:AAM3895	658[0n]
Gasteruptiidae	Gasteruption tournieri	BC ZSM HYM 05109	Germany	24-Jul-2008	SNSB, Zoologische Staatssammlung Muenchen	BOLD:AAM3895	658[0n]
Gasteruptiidae	Gasteruption tournieri	BC ZSM HYM 18414	Germany	22-Jul-2009	SNSB, Zoologische Staatssammlung Muenchen	BOLD:AAM3895	658[0n]
Gasteruptiidae	Gasteruption tournieri	BC ZSM HYM 18418	France	10-Jul-2010	SNSB, Zoologische Staatssammlung Muenchen	BOLD:AAM3895	658[0n]
Gasteruptiidae	Gasteruption tournieri	BC ZSM HYM 18420	Germany	22-Jul-2009	SNSB, Zoologische Staatssammlung Muenchen	BOLD:AAM3895	658[0n]
Gasteruptiidae	Gasteruption tournieri	ZSM-HYM-34400-E05	Croatia	08-Jul-2017	Private Collection of Petr Bogusch	BOLD:AAM3895	602[0n]
Gasteruptiidae	Gasteruption tournieri	ZSM-HYM-34400-E06	Czech Republic	14-Aug-2017	Private Collection of Petr Bogusch	BOLD:AAM3895	636[0n]
Gasteruptiidae	Gasteruption undulatum	BC ZSM HYM 05102	Germany	22-Jun-2008	SNSB, Zoologische Staatssammlung Muenchen	BOLD:AAM3894	658[0n]
Gasteruptiidae	Gasteruption undulatum	BC ZSM HYM 05103	Germany	22-Jul-2004	SNSB, Zoologische Staatssammlung Muenchen	BOLD:AAM3894	658[0n]
Gasteruptiidae	Gasteruption undulatum	BC ZSM HYM 05104	Germany	22-Jul-2004	SNSB, Zoologische Staatssammlung Muenchen	BOLD:AAM3894	658[0n]
Gasteruptiidae	Gasteruption undulatum	BC ZSM HYM 05105	Germany	22-Jul-2004	SNSB, Zoologische Staatssammlung Muenchen	BOLD:AAM3894	658[0n]
Gasteruptiidae	Gasteruption undulatum	BC ZSM HYM 18385	Germany	22-Jul-2009	SNSB, Zoologische Staatssammlung Muenchen	BOLD:AAM3894	658[0n]
Gasteruptiidae	Gasteruption undulatum	BC ZSM HYM 18386	Germany	22-Jul-2009	SNSB, Zoologische Staatssammlung Muenchen	BOLD:AAM3894	658[0n]
Gasteruptiidae	Gasteruption undulatum	BC ZSM HYM 18388	Germany	22-Jul-2009	SNSB, Zoologische Staatssammlung Muenchen	BOLD:AAM3894	658[0n]
Gasteruptiidae	Gasteruption undulatum	ZSM-HYM-34400-A11	Hungary	23-Jul-2017	Private Collection of Petr Bogusch	BOLD:AAM3894	625[0n]
Gasteruptiidae	Gasteruption undulatum	ZSM-HYM-34400-A12	Czech Republic	26-Jun-2019	Private Collection of Petr Bogusch	BOLD:AAM3894	628[4n]
Gasteruptiidae	Gasteruption undulatum	ZSM-HYM-34400-B01	Czech Republic	14-Jun-2019	Private Collection of Petr Bogusch	BOLD:AAM3894	620[0n]
Gasteruptiidae	Gasteruption undulatum	ZSM-HYM-34400-H08	Slovakia	07-Jun-2018	Private Collection of Petr Bogusch	BOLD:AAM3894	632[0n]
Gasteruptiidae	Gasteruption variolosum	ZSM-HYM-34400-G09	Greece	25-Aug-2020	Private Collection of Petr Bogusch	BOLD:AEO5161	611[0n]
Gasteruptiidae	Gasteruption variolosum	ZSM-HYM-34400-G10	Greece	25-Aug-2020	Private Collection of Petr Bogusch	BOLD:AEO5161	631[1n]
Gasteruptiidae	Gasteruption variolosum	ZSM-HYM-34400-G11	Turkey	21-Jun-2010	Oberoesterreichisches Landesmuseum, Biologiezentrum, Linz	BOLD:AEO5161	585[0n]
Stephanidae	Stephanus serrator	BC ZSM HYM 05525	Germany	15-Jul-2008	SNSB, Zoologische Staatssammlung Muenchen	BOLD:AAN9848	658[0n]
Stephanidae	Stephanus serrator	BC ZSM HYM 20590	Germany	01-Jul-2012	SNSB, Zoologische Staatssammlung Muenchen	BOLD:AAN9848	664[0n]

Family	Species	Specimen ID	Country	Collection Date	Depository	BIN	COI-5P
Stephanidae	Stephanus serrator	BC ZSM HYM 24740	Germany	19-Jul-1994	SNSB, Zoologische Staatssammlung Muenchen	BOLD:AAN9848	658[0n]
Stephanidae	Stephanus serrator	BC ZSM HYM 24741	Germany	22-Jun-2008	SNSB, Zoologische Staatssammlung Muenchen	BOLD:AAN9848	658[0n]
Trigonalidae	Pseudogonalos hahnii	GBOL 03178	Germany	05-Jun-2010	SNSB, Zoologische Staatssammlung Muenchen	BOLD:ACL1363	657[0n]
