# Supplementary material for: ﻿DNA Barcoding of Central European Gasteruptiidae and the rarely-collected families Evaniidae, Stephanidae, Trigonalidae, and Aulacidae (Hymenoptera, Apocrita)
Source: Zookeys. 2024 Jan 17;1189:275–86. doi: 10.3897/zookeys.1189.114478 (PMC10809266; doi:10.3897/zookeys.1189.114478)
Supplement: Supplementary material 2 — Barcoding statistics [file zookeys-1189-275_article-114478__-s002.pdf]

Supplement 2: Barcoding statistics with mean intraspecific distance, maximum intraspecific distance, nearest neighbour species, distance to nearest neighbour species, Barcode Index Number (BIN), country, and number of specimens. Asterisks indicate species with BIN sharing.

Family	Species	Mean	Max	Nearest species	NN distance	BIN	Country	Specimens
**Aulacidae**	Aulacus striatus	0.18	0.31	Gasteruption merceti	23.48	BOLD:ABU9057	Netherlands	4
	Pristaulacus compressus	N/A	0	Gasteruption nigritarse	28.18	BOLD:ADA3999	France	1
**Evaniidae**	Brachygaster minutus	0.72	1.08	Gasteruption jaculator	30.68	BOLD:AAW7612	Germany	2
							Russia	1
**Gasteruptiidae**	Gasteruption assectator	0.15	0.72	Gasteruption boreale	0	BOLD:AAM3893*	France	2
							Germany	14
							Hungary	1
							Slovakia	2
	Gasteruption boreale	0.16	0.36	Gasteruption assectator	0	BOLD:AAM3893*	Czech Republic	4
							Slovakia	1
	Gasteruption caucasicum	0.59	1.13	Gasteruption phragmiticola	9.24	BOLD:AAM4594	Croatia	1
							Czech Republic	1
							Germany	3
							Hungary	1
							Italy	2
							Slovakia	1
	Gasteruption diversipes	N/A	0	Gasteruption schlettereri	0	BOLD:AEO5656*	Slovakia	1
	Gasteruption dolichoderum	0.76	0.82	Gasteruption undulatum	9.73	BOLD:ACE0224	Cyprus	2
							Turkey	1
	Gasteruption erythrostomum	1.28	3.39	Gasteruption insidiosum	3.56	BOLD:AAN0587	Germany	2
						BOLD:AEO6078	Austria	1
							Czech Republic	2
							Germany	4
	Gasteruption forticorne	0.18	0.26	Gasteruption schlettereri	8.91	BOLD:AED0665	Croatia	1
							Hungary	1
							Slovakia	1
	Gasteruption foveiceps	0.19	0.19	Gasteruption nigrescens	2.96	BOLD:AAM4590	Italy	2
	Gasteruption freyi	0	0	Gasteruption boreale	8.47	BOLD:AEN9285	Austria	1
							Czech Republic	1

	Gasteruption goberti	0.32	0.32	Gasteruption opacum	9.33	BOLD:AEO5240	Greece	1
							Turkey	1
	Gasteruption hastator	1.17	2.63	Gasteruption freyi	9.91	BOLD:AAV6833	Czech Republic	1
							France	1
							Hungary	2
							Slovakia	1
						BOLD:AEZ6954	Italy	1
	Gasteruption hungaricum	0	0	Gasteruption subtile	7.22	BOLD:AEO6155	Hungary	1
							Slovakia	1
	Gasteruption insidiosum	8.06	8.06	Gasteruption erythrostomum	3.56	BOLD:AEO1542	Turkey	1
						BOLD:AEO1543	Slovakia	1
	Gasteruption jaculator	1.34	3.28	Gasteruption subtile	6.55	BOLD:AAN0586	Germany	4
							Czech Republic	1
							Slovakia	1
	Gasteruption laticeps	1.20	2.58	Gasteruption diversipes	8.63	BOLD:AAU2086	Czech Republic	4
							Greece	1
							Hungary	1
							Slovakia	1
						BOLD:ACH3937	France	2
							Italy	1
	Gasteruption merceti	0.57	1.47	Gasteruption variolosum	9.10	BOLD:ACE0348	Czech Republic	1
							Italy	4
							Serbia	1
							Slovakia	1
	Gasteruption minutum	1.13	2.62	Gasteruption assectator	6.88	BOLD:ACE0418	Czech Republic	2
							France	1
							Italy	2
							Slovakia	1
	Gasteruption nigrescens	0.14	0.22	Gasteruption foveiceps	2.96	BOLD:AAM4589	Czech Republic	1
							Germany	2
							Slovakia	2
							Slovakia	1
	Gasteruption nigritarse	0.41	0.76	Gasteruption assectator	0	BOLD:AAM3893	Czech Republic	5

							Hungary	1
							Slovakia	1
	Gasteruption opacum	0.81	1.88	Gasteruption schlettereri	9.06	BOLD:ACH4090	Croatia	2
							Hungary	1
							Italy	3
							Slovakia	1
	Gasteruption paternum	3.40	5.11	Gasteruption schlettereri	11.51	BOLD:ACE0342	Italy	2
						BOLD:ACE0475	Austria	1
	Gasteruption phragmiticola	0.21	0.48	Gasteruption insidiosum	8.53	BOLD:AAM5488	Czech Republic	2
							Germany	2
							Hungary	3
	Gasteruption schlettereri	0.14	0.24	Gasteruption diversipes	0	BOLD:AEO5656*	Croatia	1
							Italy	2
	Gasteruption subtile	0.30	0.30	Gasteruption jaculator	6.55	BOLD:ADD2731	Slovakia	2
	Gasteruption tournieri	0.91	1.55	Gasteruption diversipes	9.99	BOLD:AAM3895	Croatia	1
							Czech Republic	1
							France	1
							Germany	6
	Gasteruption undulatum	0.62	1.31	Gasteruption assectator	8.13	BOLD:AAM3894	Czech Republic	2
							Germany	7
							Hungary	1
							Slovakia	1
	Gasteruption variolosum	0.35	0.52	Gasteruption merceti	9.10	BOLD:AEO5161	Greece	2
							Turkey	1
**Stephanidae**	Stephanus serrator	0	0	Gasteruption dolichoderum	29.58	BOLD:AAN9848	Germany	4
**Trigonalidae**	Pseudogonalos hahnii	N/A	0	Gasteruption laticeps	24.26	BOLD:ACL1363	Germany	1
